# Selective attention to question-relevant text information precedes high-quality summaries: Evidence from eye movements

**DOI:** 10.16910/jemr.12.1.6

**Published:** 2019-05-30

**Authors:** José A. León, José David Moreno, Inmaculada Escudero, Johanna K. Kaakinen

**Affiliations:** Universidad Autónoma de Madrid, Spain; UNED, Madrid, Spain; University of Turku, Finland

**Keywords:** Summary quality, strategic selective processing, eye movements, reading comprehension, individual differences

## Abstract

Comprehension and summarizing are closely related. As more strategic and selective processing during reading should be reflected in higher quality of summaries, the aim of this study was to use eye movement patterns to analyze how readers who produce good quality summaries process texts. 40 undergraduate students were instructed to read six expository texts in order to respond a causal question introduced in the end of the first paragraph. After reading, participants produced an oral summary of the text. Based on the quality of the summaries, participants were divided into three groups: High, Medium and Low Quality Summaries. The results revealed that readers who produced High Quality Summaries made significantly more and longer fixations and regressions in the question-relevant parts of texts when compared to the other two summary groups. These results suggest that the summary task performance could be a good predictor of the reading strategies utilized during reading.

## Introduction

Current theories of text comprehension assume that readers try to form a coherent memory representation of the text they read [ 1 ]. Forming a coherent memory representation of the text requires that the reader is capable of connecting the ideas presented in the text to each other as well as of integrating text information with his or her background knowledge [ 2, 3 ]. According to the Lansdscape model [ 4, 5 ], the memory representation of text reflects the landscape of activations of different concepts during the course of reading: concepts may be activated automatically by the information presented in text [ 6 ] or they could be retrieved from the episodic text representation or long-term memory [ 4, 5 ]. Concepts that are simultaneously activated are likely to be connected with each other and to be encoded to memory.

The goal or the task the reader has in mind plays an important role in how a reader processes and learns information presented in texts [ 7 ]. Goals are formed on the basis of reader’s personal interests and intentions, or they could be induced by instructions given to the reader [ 7, 8, 9 ]. The task the reader is trying to accomplish has a big impact on how much effort reader invests in building a coherent memory representation of text [ 10, 11, 12, 13, 14 ].

The task the reader has in mind defines what information is considered important in text: when readers are given specific instructions for reading, they rate text paragraphs containing task-relevant information as more important than paragraphs containing irrelevant information [ 15 ]. Text information is then processed in order to meet the reading goal, and relevant information is given priority over information that is not relevant. For example, when readers are presented with a question prior to reading, they will devote more attention to question-relevant than question-irrelevant text contents, and question-relevant information is more likely to be encoded to memory and remembered [ 16 ]. Previous eye tracking studies show that readers invest more processing time on relevant than on irrelevant text information, and these effects can be seen during the first-pass reading as well as in later look-backs to relevant text segments [ 17, 18, 19, 20, 21 ]. After reading, readers show better recall of task-relevant than irrelevant information [ 22, 23, 24 ]. In summary, when readers have a specific reading task in mind, they consider task-relevant text segments as important, and direct processing resources selectively to relevant text information. The resulting memory representation of text reflects the selective attention paid to the relevant text segments: recall is better for task-relevant than irrelevant information.

Prereading questions are an example of a specific reading task that efficiently guides readers’ attention to certain information in text and improves memory for it [ 25, 26, 27, 28, 29, 30, 31 ]. Lewis and Mensink [ 26 ] conducted two experiments in order to test the benefits of prereading questions for increasing attention to and recall of relevant sentences in texts. Using eye tracking, they found that participants directed additional attention, as reflected in increased first-pass and look-back times, to the relevant sentences in the texts. Participants also produced more information related to the relevant sentences in their recalls if they were exposed to the prereading questions.

In previous studies on task effects, memory for text information has mainly been measured with a recall task, in which participants have been asked to write down as many main points presented in text as they can after reading [ 17, 18, 21 ]. While free recall reflects the type of information the reader has stored in memory, it is not necessarily the optimal way to measure comprehension [ 32 ]. Another, perhaps better way would be to ask readers to summarize the contents of the text [ 33, 34, 35, 36, 37, 38, 39, 40, 41, 42 ]. When readers summarize a passage, they have to identify and express the core concepts that represent the general themes of the text in a coherent way. A summary task encourages deep understanding of the text because it requires active construction of the meaning as opposed to merely choosing one response from several alternatives (as in multiple-choice questions), answering isolated questions (as in open-ended questions), or simply reproducing information read in text (as in free recall). Synthesis and coherence are two key aspects of a good summary. In the present study, we used an oral summary task in order to measure how readers synthesize and form a coherent representation of the text they read. An oral summary is a concise production about the most important information of the text, and it is a more natural and spontaneous output than a written summary. Written summaries require increased attention to grammatical correctness and writing style, so typically more time is needed to plan and produce a written response. Thereby, producing summaries orally minimizes possible interference from writing-specific requirements (e.g., planning activities, attention to grammatical correctness, writing style), and thus an oral summary can be considered as a purer indicator of the quality of the memory representation than a written summary.

But what makes a good summary? Several models to evaluate the quality of summaries have been proposed. Some authors, such as Kirkland and Saunders [ 36 ], suggest that summaries for expository texts should be evaluated on the basis of several criteria: (a) the summary provides a general overview of the text and emphasizes the relations existing between the main ideas, (b) the information given is clarified by secondary ideas, (c) the summarizer makes clear when original text is used, and (d) the summarizer uses his or her own words. In the summary analysis model of Jorge and Kreis [ 35 ], the authors identify several parameters to measure the quality of summaries: cohesion and coherence, inclusion of the main ideas contained in the source text, conciseness, information about the source text, and absence of personal opinion.

We propose that the quality of summaries for expository texts can be described with two main dimensions: content and coherence [ 43 ]. Content criteria concern the extent to which the summary reflects the essential content of the text, such as whether the summary includes the most task-relevant ideas presented in the text or not. Coherence criteria refer to the connections built between idea units presented in text as well as with reader’s knowledge base, including causal relations between the relevant ideas. These dimensions reflect the extent to which the reader has adopted task-relevant core content of the text, integrated it with his or her prior knowledge, as well as constructed the causal relations between the relevant ideas of the text, including reasons and consequences. All of these aspects should be clear and explicit in a good summary. These criteria have been proved to be useful for assessing reading comprehension [ 33, 34, 38, 40 ]. The reliability and validity of a summary test, which is based on scoring the content and coherence of summaries, has been shown to be good [ 37, 44, 45, 46, 43, 47 ]. For example, León et al. [ 47 ] demonstrated that a summary test (RESUMeV), in which the quality of the summaries is scored for content and coherence, has high reliability (interrater *r*’s .69-.97, for four independent raters). León, Escudero, Olmos, Moreno and Martín [ 48 ] showed that the summary task scores correlate highly (*r* = .82) with multiple choice test performance, indicating that the summary task is a valid measure of reading comprehension. Additionally, León et al [ 46 ] analyzed the causal network in a narrative text and compared this to the causal networks generated by the students in their written summaries of the original text. The results showed that more competent readers produced more causal nodes in their summaries and thus got higher scores for causal coherence than less competent readers, which demonstrates the criterion validity of the summary task.

The crucial question is how readers who produce coherent summaries containing relevant information actually process the text information? In order to answer this question, we need to examine the moment-to-moment processes as they occur during reading. Eye tracking is a fruitful methodology for examining the cognitive processes occurring during reading [ 49, 50, 51 ], and it can be used to identify reading strategies that underlie successful expository text comprehension [ 52 ]. For example, Hyönä and colleagues [ 52 ] asked college-age students to read two expository texts for comprehension. Readers’ eye movements were recorded during the course of reading, and after reading participants wrote a free recall of the texts they read. The analysis of the eye movement data revealed four different reader groups, who also differed with respect to the comprehension of the text as reflected in the quality of their recall protocols. ‘Topic structure processors’, who were sensitive to the topic structure of the text and made eye fixations back to the subheadings and topic sentences especially from the end of the paragraphs, showed good recall of the text contents. Also ‘Fast linear readers’, who progressed in text relatively quickly and did not make look-back fixations, gained good memory of the text. These results show that the pattern of eye movements is informative about the comprehension processes: topic structure processors invest extra effort in building links between text elements, whereas fast linear readers encode text information to memory relatively effortlessly.

In the present study, we used eye tracking to examine individual differences in how readers manage the demands of a specific reading task – answering a question presented in the beginning of the text – and specifically, whether readers who produce high quality summaries utilize different processing strategies than readers whose summaries are not as comprehensive. We were particularly interested in how readers who provided comprehensive summaries inspected question-relevant and –irrelevant text information.

Individual differences in general cognitive abilities, such as working memory capacity, play a crucial role in how readers process and comprehend text [ 53 ]. Previous eye movement studies show that there are individual differences in how adult readers inspect and recall task-relevant and irrelevant text information [ 23, 20 ]. However, these prior studies have not examined differences in the quality of the memory performance, such as both the content and coherence of the recalls. Moreover, prior studies have used written recalls, which may not be optimal for measuring comprehension of text, as discussed above. It is important to understand how different readers perform when given a specific reading task, as it is a way of understanding the performance differences between participants in modern reading assessments such as the OECD-PISA and PIAAC studies [ 54, 55 ]. What the present study adds to the current literature is thus important knowledge about what kind of processing strategies are successful and result in good text comprehension, as measured by an offline summary task.

The texts used in the present study were short expository texts consisting of three paragraphs: an introduction, which also presented a question related to the text’s topic, a paragraph providing information relevant for answering the question presented in the introduction, and a paragraph containing information related to the topic of the text but not relevant to answering the question. For example, one of the texts introduced history related to the pollution of river Thames. In the introduction, general overview about history was given, and in the end of the introduction, a question was presented (“But how did the river become so contaminated?”). In the question-relevant paragraph, reasons for why river Thames got polluted were discussed. In the question-irrelevant paragraph, consequences of the river’s pollution were given. This information, even though it was highly related to the overall topic of the text, was not relevant for answering the question presented in the introduction.

After reading the texts, participants provided oral summaries. Summaries were rated for the content (i.e. whether the summary contained information relevant to answering the question) and coherence, according to the scheme proposed by León and Escudero [ 43 ]. There was individual variability in the quality of the summaries, and participants were divided into three groups on the basis of the quality of their summaries: low, medium, and high quality summary groups. We then compared the eye movement patterns of the reader groups. Following Hyönä, Lorch & Rinck [ 49 ], we computed measures that reflect the initial processing of the text paragraphs ( *first-pass reading time*) and the *number of returns* and the *duration of look-backs* made to the paragraphs from subsequent parts of text, in addition to the total time spent reading the paragraph.

On the basis of previous eye-tracking studies on expository text reading [ 52, 24, 21, 25 ], we expected that there are consistent individual differences between readers in the processing strategies they use while they are reading. Previous research suggests that some readers who are more sensitive to topic relevant information tend to use a selective reading strategy, in which they devote additional resources to this information and make frequent rereading and looking back to the relevant sections of the text in order to integrate information in memory. On the other hand, some readers are less sensitive to topic relevant information and present a non-selective reading strategy, in which they do not dedicate additional attention to topic relevant information. We expected that readers who produce good quality summaries would present a selective reading strategy, which would be reflected in eye movement records as longer fixation times to question-relevant than to question-irrelevant text segments, already during initial reading of the paragraph. Moreover, we expected that these readers would demonstrate more looking back to the introductory paragraph from the relevant paragraph, as integrating the information presented later in the text with the paragraphs that contains a question should benefit the construction of a coherent memory representation.

## Methods

### Participants

Participants were 40 university students (12 males; age range: 20–23 years) enrolled at a public Spanish university. All participants were third-year psychology majors who volunteered to participate in the experiment to get an extra course credit. All participants were native speakers of Spanish (the language studied here), and had normal or corrected-to-normal vision.

### Apparatus

Eye movement data were collected with an EyeTech™ Digital Systems VT2 infrared eye tracker, with a sampling rate of 80 Hz. The VT2 has two infrared light sources and an integrated infrared camera. The camera was attached under the screen of a 15-inch laptop computer, which was used for the presentation of the texts. The eye tracker connects via USB to a Windows computer and captures the eye gaze location (x, y coordinates). Registration of eye movements was binocular and in the case that it was not possible, monocular. A chin-and-forehead rest was used to stabilize the head position during the test. The screen of the laptop was placed at 60 cm from the participant, and it worked with a 100 Hz refresh rate and a 1366 x 768 resolution. Following the calibration standards of the manufacturer, the 97% of the calibrations made for this study were considered “excellent” and the 3% “very good”.

### Materials

Eight expository texts were created for this experiment to be used as stimuli; two texts were used as practice materials. The experimental texts introduced eight different topics (the Thames, Mediterranean diet, the suitcase evolution, popcorn history, urban growth, detective novel, insomnia and the greenhouse effect). Each text consisted of three paragraphs (an example text is presented in Appendix A). The first paragraph was an introductory paragraph, which always finished with a question related to the main topic of the text. The topic was then developed in two paragraphs: one including information relevant to the question presented in the end of the introductory paragraph, and the other containing information that was relevant to the topic of the text, but irrelevant to answering the question. The texts were 200-250 words long.

We applied the updated Dale-Chall readability formula [ 56 ] for each text in order to ensure that the texts presented in the experiment were appropriately challenging for the range of participants tested. The formula gives an approximate estimate of the academic grade level that a reader needs in order to understand a text. A score around 9 means that the text should be appropriately understood by college students, and scores below 9 indicate that the text is easier (i.e. does not require college-level reading abilities). The scores for the texts were: the Thames, 8.3; Mediterranean diet, 9.82; the suitcase evolution, 8.96; popcorn history, 6.52; urban growth, 9.49; detective novel, 10.19; insomnia 8.56; the greenhouse effect, 8.98.

There were two versions of each text: one in which the question-relevant paragraph was presented after the introduction, and another one in which the question-irrelevant paragraph came after the introduction. Each participant saw only one version of each text, and the location of the question-relevant paragraph was counterbalanced across participants. Thus, each participant saw three texts where the relevant paragraph was the first paragraph after the introduction and three texts where it was the second. The two text versions of each text were presented equally often across participants.

The texts were presented one at a time on a computer screen, with a maximum of 14 lines of text on one screen. Texts were presented in Times New Roman font, with a font size of 12, and 2 points of line spacing. Participants were allowed to freely view the text for as long as they needed.

### Relevance ratings

We conducted a norming study in order to verify that particular paragraphs are more relevant than others with respect to the task instructions given to the participants. Fifteen participants (3^rd^ year psychology students) who did not participate in the actual experiment volunteered to get an extra course credit. Participants were presented with the instructions used in the actual experiment, and asked to select the paragraphs they thought were relevant with respect to the instructions. Each participant rated each of the six experimental texts. The consistency in ratings was very high: 97.8% of the given ratings overlapped with our pre-set definition of relevance. In only 2.2% of the responses the introductory paragraph was rated as the most relevant; it is worth highlighting that none of the responses indicated the irrelevant paragraph as the most relevant of the text.

### Eye movement measures

Before running the linear mixed effects models to analyze the data, the following measures were computed separately for the paragraphs introducing question-relevant and irrelevant information: *the total fixation time, first-pass reading time, look-back duration, number of returns to the introductory paragraph and duration of look-backs to the introductory paragraph.* The total fixation time was the total time spent reading the paragraph. First-pass reading time was the summed duration of fixations made to the paragraph during the first-pass reading of it. Look-back duration is the summed duration of fixations returning back to the paragraph after the reader has viewed other parts of text. For the introductory paragraph two measures were computed: the number of returns, and the duration of look-backs to the introductory paragraph. The number of returns is the number of times that the reader returned to the introductory paragraph from subsequent parts of text, and the look-back duration is the summed duration of fixations made during these returns. As the number of fixations and duration measures were very highly correlated, only duration measures will be reported here. The eye tracking technology used in the present study only allowed us to analyze eye movements on a paragraph level, not on the sentence or word levels. However, taking into account the purpose of the present study, paragraph level is sufficient for describing the global reading strategies utilized by participants.

### Scoring of the summaries

Each participant generated an oral summary after reading each of the six experimental texts. The summaries were recorded and transcribed, and then scored on the basis of two criteria: *content and coherence* [ 43, 47 ]. Each text was evaluated by three independent raters on a 5-point scale (0-4 points); the inter-rater agreement (Cohen´s Kappa) ranged from .68 to .94. The score reflects both the content of the summary (whether the reader had correctly identified and represented relevant main idea in the summary), and also the coherence of the summary (causal connections that were established between ideas). To exemplify this, the coding scheme to score the summaries of one of the experimental texts is presented in Appendix A. The scores of the three raters were averaged for each text, and a mean summary score for each participant was computed. On the basis of the percentiles of their summary scores, the participants were divided into three different groups of equal size: High Quality Summary (HQS) group, Medium Quality Summary (MQS) group and Low Quality Summary (LQS) group. Examples of summaries produced by participants from different summary groups are presented in Appendix A.

In addition to the summary scores, we computed the number of words in the protocol that corresponded to the sentences presented in the text. This measure was thought to reflect the quantity of information retained from the texts. Word counts were computed separately for the introductory paragraph, the relevant paragraph, and the irrelevant paragraph. Two independent raters scored 30 randomly selected recall protocols; inter-rater reliability was high (92%, Cohen’s Kappa = .83), and the rest of the protocols were rated by only one rater.

### Procedure

In the beginning of the experimental session, the eye tracker was calibrated with a 16-point calibration scheme. Calibration was repeated after every two texts. Participants were instructed to read the texts so that they would be able to summarize the main contents, and especially to answer the question presented in the text. The exact instructions given to the participants can be found in the Appendix B. Two practice trials preceded the first experimental text to adjust the participants to the eye-tracking equipment. After reading each text, participants produced an oral summary of the text; the protocols were recorded. The experimental session took approximately 20 minutes per participant.

## Results

### Data preparation and statistical analyses

Participants were divided into three equally sized groups on the basis of the percentiles of their mean summary scores: *High Quality Summary* (HQS) group (n=14, scores 3 -- 4 points), Medium quality summary (MQS) group (n=13, scores 2.16 -- 2.88 points), and low quality summary (LQS) group (n=13, scores 1.16 -- 2 points).

The data were analyzed with linear mixed effects models using the lme4 package (version lme4_1.1-12) [ 57 ] for R statistical software (version 3.3.2) [ 58 ]. Separate models were fitted for each dependent measure: *total fixation time, first-pass reading time, look-back duration, number of returns to the introductory paragraph, duration of look-backs to the introductory paragraph, and summary task performance (word count)*. As the number of fixations and duration measures were very highly correlated ( *r’s* .98 - .99), only duration measures are reported here. Summary Group, Relevance and their interaction term were entered as fixed effects to the models of eye-tracking measures. Summary Group and Relevance were dummy coded, LQS group and irrelevant paragraph were the baseline. For the model of summary task performance (word count), Summary group and Paragraph (Introductory, Relevant or Irrelevant) were entered as dummy coded fixed effects, LQS and Introductory paragraph as a baseline. Random intercepts for participants and items (i.e. texts) were included in the random part of the models. The models were run using non-transformed data. The main reason to proceed in this way was that no transformations were needed, as the normal probability plots indicated that the distributions of the dependent measures were not severely skewed.

Significant interactions were followed up by computing simple slopes for each summary group. |T|-values > 1.96 were considered to indicate a statistically significant effect.

Observed means and standard deviations for all eye movement measures as a function of the quality of the summary and relevance are presented in Table 1. Tables for random effects and estimates of fixed effects for all depended measures are presented in Appendix C.

**Table 1:** Means and standard deviations for the eye tracking measures as a function of relevance and summary group.

Summary group							
		LQS		MQS		HQS	
Measure	Paragraph	M	SD	M	SD	M	SD
*Total*	Relevant	8.81	5.81	8.98	6.34	17.14	10.11
	Irrelevant	5.75	4.73	5.00	3.21	7.17	4.96
*1^st^ pass*	Relevant	6286	4470	6837	4762	8905	6232
	Irrelevant	5375	4485	4563	3063	5756	3696
*Look-backs*	Relevant	2522	3557	2148	3880	8232	9363
	Irrelevant	376	1983	437	1830	1416	3396
*Returns*	Relevant	.61	.88	.69	.90	1.46	1.57
	Irrelevant	.35	.68	.35	.56	.48	.69
*Look-backs to intro*	Relevant	753	2885	577	1189	1757	2254
	Irrelevant	892	2971	867	2847	833	2241.2

LQS= low quality summary, MQS= medium quality summary, HQS= high quality summary, Total = total fixation time (s); 1st pass = first-pass reading time (ms); Look-backs = look-back duration (ms), Returns = number of returns to the introductory paragraph, Look-backs to intro = duration of look-backs to the introductory paragraph (ms).

### Total fixation time

**Figure 1.**
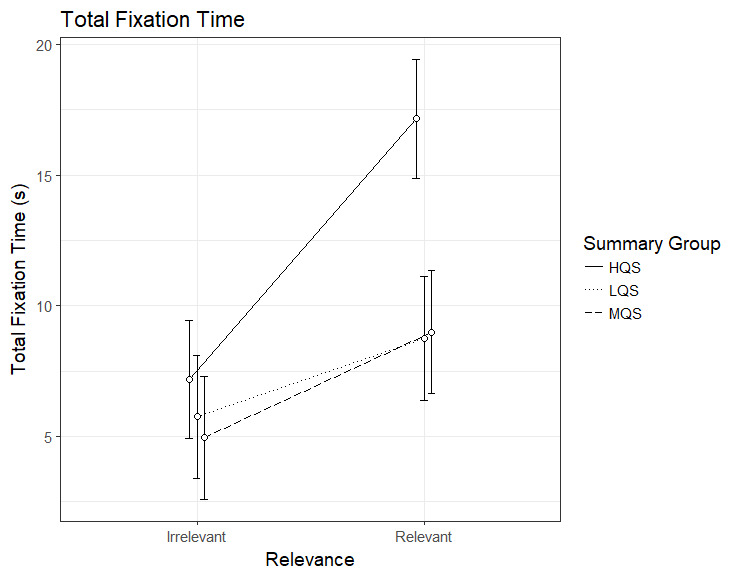
Model estimates for total fixation time on relevant and irrelevant paragraphs as a function of summary group. LQS=low quality summary, MQS=medium quality summary, HQS=high quality summary. Error bars represent 95% CI’s.

The analysis of the total fixation time revealed an interaction between Summary Group (high) and Relevance, b=6.98, 95%CI [4.78 – 9.18], *t*=6.21. As can be seen in Figure 1, the HQS group showed a sizable relevance effect, i.e. longer fixation time on relevant than irrelevant paragraphs, b=9.98, 95%CI [8.45 – 11.50], *t*=12.80. Also the MQS group, b=4.03, 95%CI [2.44 – 5.62], *t*=4.98, and the LQS group, b=3.00, 95%CI [1.41 – 4.58], *t*=4.78, showed a relevance effect but it was clearly smaller than that for the HQS group. Looking at the figure, it is evident that the difference between the groups is in the time spent on relevant paragraphs: HQS group demonstrates much longer fixation time on relevant paragraphs in comparison to the two other groups.

### First-pass reading time

**Figure 2.**
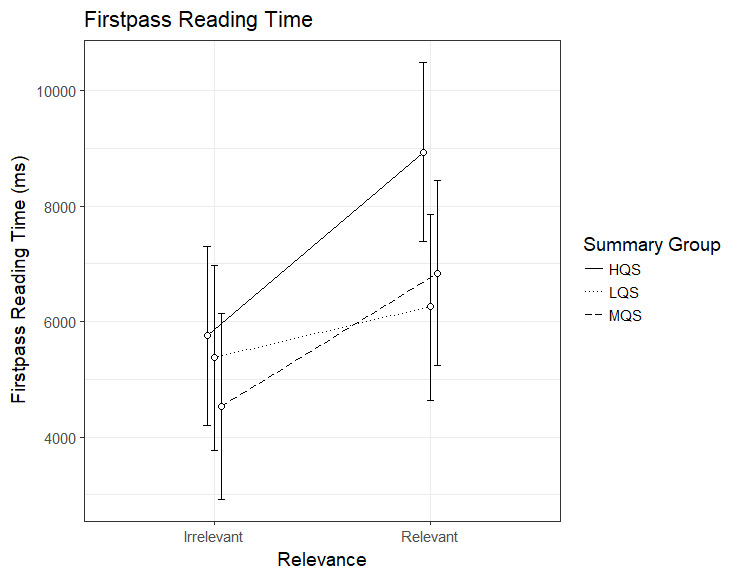
Model estimates for first-pass reading time for relevant and irrelevant paragraphs as a function of summary group. LQS=low quality summary, MQS=medium quality summary, HQS=high quality summary. Error bars represent 95% CI’s.

The analysis of the first-pass reading time revealed an interaction between Summary Group (high) and Relevance, b=2306.8, 95%CI [611.49 – 4002.20], *t*=2.67. As can be seen in Figure 2, the HQS group showed a clear relevance effect, b=3178.40, 95%CI [2002.13 – 4354.59], *t*=5.30. The difference between relevant and irrelevant paragraphs was smaller, yet significant, in the MQS group, b=2303.7, 95%CI [1082.80 – 3524.66], *t*=3.70. However, there was no evidence for a relevance effect in the LQS group, b=871.5, 95%CI [-349.42 – 2092.45], *t*=1.40. The interaction seems to be driven by the HQS group spending longer first-pass reading time than the other groups on relevant paragraphs.

### Look-back duration

**Figure 3.**
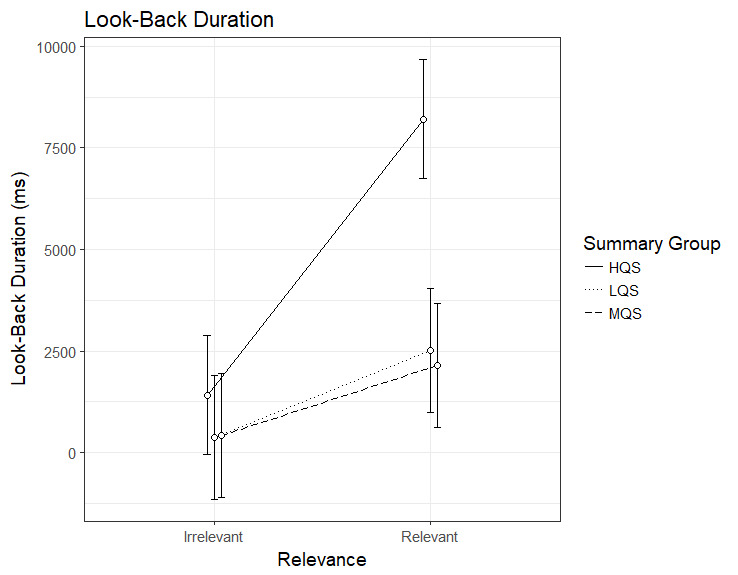
Model estimates for look-back duration for relevant and irrelevant paragraphs as a function of summary group. LQS=low quality summary, MQS=medium quality summary, HQS=high quality summary. Error bars represent 95% CI’s.

**Figure 4.**
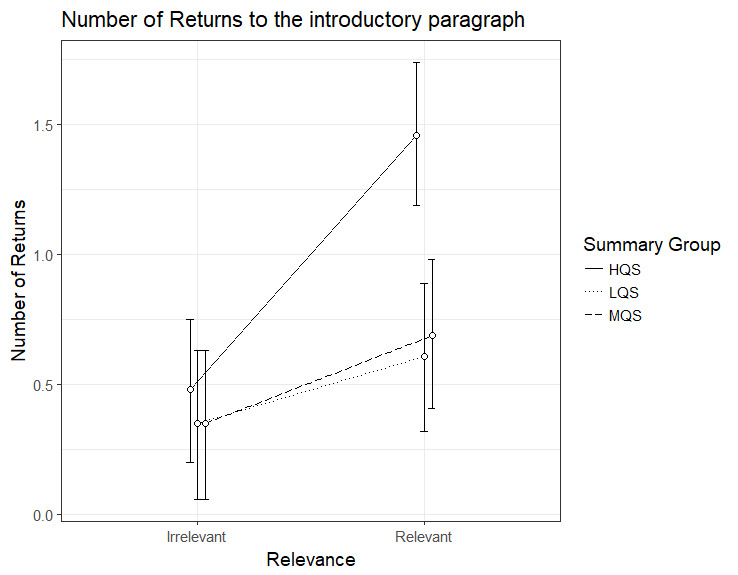
Model estimates for number of returns to the introductory paragraph from relevant and irrelevant paragraphs as a function of summary group. LQS=low quality summary, MQS=medium quality summary, HQS=high quality summary. Error bars represent 95% CI’s.

The analysis of look-back duration showed an interaction between Summary Group (high) and Relevance, b=4658.45, 95%CI [2810.38 – 6506.53], *t*=4.94. As can be seen in Figure 3, the HQS group showed a clear relevance effect, b=6792.1, 95%CI [5509.93 – 8074.32], *t*=10.38, indicating that readers spent longer time looking back to relevant than irrelevant paragraphs. So did the MQS group, b=1723.85, 95%CI [392.93 – 3054.78], *t*=2.54, and the LQS group, b=2133.68, 95%CI [802.75 – 3464.60], *t*=3.14, indicating that even though all three groups spent longer time looking back to relevant than irrelevant paragraphs, the effect was greater in the HQS than in the other groups. As is apparent from Figure 4, the interaction seems to be driven by the HQS group spending longer time looking back to relevant paragraphs than the two other groups.

### Number of returns to the introductory paragraph

The results for the number of returns to the introductory paragraph showed an interaction between Summary Group (high) and Relevance, b=.72, 95%CI [.34 – 1.10], *t*=3.76. As can be seen in Figure 4, the HQS group showed a clear relevance effect, b=.98, 95%CI [.72 – 1.24], *t*=7.35, indicating that they did more returns to the introductory paragraph from the relevant than irrelevant paragraphs. This was the case also for the MQS group, b= 0.34, 95%CI [.07 - .61], *t*=2.49; for the LQS group the effect just failed to reach significance: b=.26, 95%CI [-.008 - .53], *t*=1.90. Again, the HQS group differed from the two other groups by making more returns from relevant paragraphs.

### Duration of look-backs to the introductory paragraph

**Figure 5.**
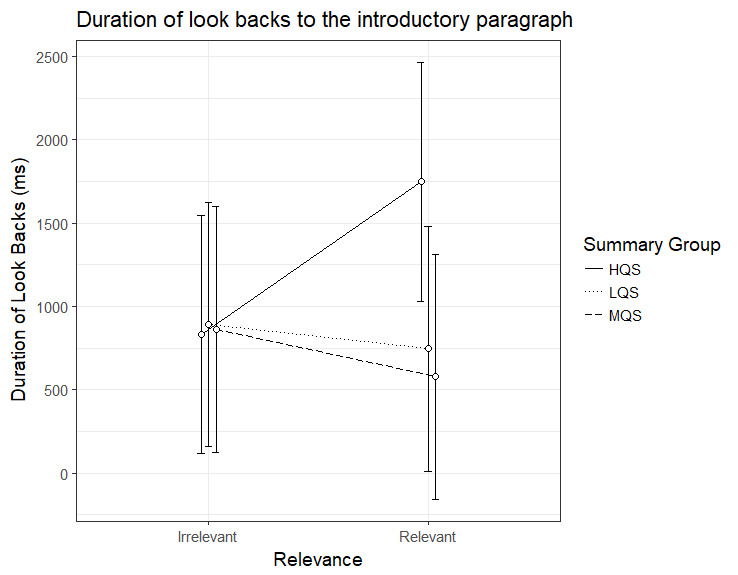
Model estimates for duration of look-backs to the introductory paragraph for relevant and irrelevant paragraphs as a function of summary group. LQS=low quality summary, MQS=medium quality summary, HQS=high quality summary. Error bars represent 95% CI’s.

The results for the duration of look-backs to the introductory paragraph showed again an interaction between Summary Group (high) and Relevance, b=1062.67, 95%CI [52.09 – 2073.24], *t*=2.06. As can be seen in Figure 5, the HQS group showed a clear relevance effect, b=915.80, 95%CI [214.66 – 1616.93], *t*=2.56, *t*=5.30, indicating that these participants made longer look-backs from relevant than irrelevant paragraphs. On the other hand, there was no indication of a relevance effect for the MQS group, b=-283.7, 95%CI [-1011.46 – 444.11], *t*=-.76, or the LQS group, b=-146.87, 95%CI [-874.65 – 580.91], *t*=-.40. As is evident from Figure 5, the HQS group participants differed from the two other groups in that they made longer look-backs from relevant paragraphs.

### Summary task performance (word count)

**Figure 6.**
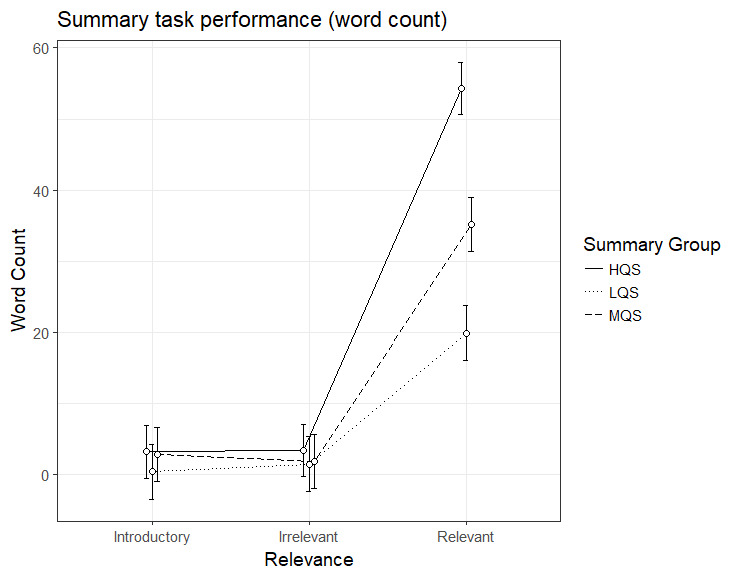
Model estimates for summary task performance (word count) for relevant, irrelevant and introductory paragraphs as a function of summary group. LQS=low quality summary, MQS=medium quality summary, HQS=high quality summary. Error bars represent 95% CI’s.

Finally, we analyzed the number of words retrieved from different parts of text (introductory, irrelevant and relevant paragraphs); the descriptive statistics are presented in Table 2. The results showed a significant interaction effect between Summary Group (high) and Relevance (relevant paragraph), b=31.55, 95%CI [26.74 – 36.37], *t*=12.84. All the three summary groups demonstrated higher recall of words presented in relevant than introductory paragraphs, HQS, b=51.06, 95%CI [47.76 – 54.37], *t*=30.27, MQS, b=32.29, 95%CI [28.87 – 35.72], *t*=18.51, and LQS, b=19.51, 95%CI [16.01 – 23.01], *t*=10.93. The relevance effect (relevant paragraph) was greatest in the HQS group, and smallest in the LQS group. As can be seen in Figure 6, participants in HQS group produced significantly more words from relevant paragraphs in their summaries than participants in the other two groups.

**Table 2:** Means and standard deviations for the summary task performance (word count) measure as a function of relevance and summary group.

Summary group						
	LQS		MQS		HQS	
Paragraph	M	SD	M	SD	M	SD
Introductory	.28	2.43	2.88	9.94	3.21	10.24
Irrelevant	1.37	5.70	1.87	6.86	3.38	10.25
Relevant	19.80	11.66	13.36	20.03	54.34	20.13

LQS= low quality summary; MQS= medium quality summary; HQS= high quality summary.

## Discussion

The present study examined how readers who produce good quality summaries process text information during the course of reading. The results showed that readers who demonstrate good summarization skills show strategic reading behavior [ 52 ] and utilize a *selective processing strategy* [ 24, 20, 25 ]: they spend more time reading question-relevant than question-irrelevant paragraphs, and this preference is seen already during the first-pass reading of the relevant paragraphs. Readers who produce high quality summaries also do longer look-backs to the relevant paragraphs. Importantly, they also make more frequent and longer look-backs to the introductory paragraph from the paragraph introducing relevant information than other readers, implying that they engage in building links between the paragraph that includes the question and question-relevant text information. This selective attention to relevant paragraphs and increased looking back to the introductory paragraph is not related to increased recall of text information in general – rather, it is related to increased recall of question-relevant text materials. These results indicate that a successful summary strategy is characterized by increased attention to relevant paragraphs and by coherence-building rereading of the introductory paragraph containing the question.

It should be noted that the differences between the summary groups were observed in the processing of question-relevant text information, not in how readers directed processing time to irrelevant paragraphs. Moreover, readers who produced poorer summaries demonstrated weaker relevance effects overall, and the group producing lowest quality summaries failed to demonstrate sensitivity to relevance during first-pass reading. These findings indicate that readers who demonstrate good summary skills are better in recognizing relevant information, and are aware of and utilize processing strategies that increase its encoding to memory.

In addition, the rating of the quality of the summaries on the basis of their content and coherence [ 43, 47 ] proved to be valid in the sense that it differentiated readers who demonstrated different processing strategies. Readers who produced more elaborated and detailed summaries that included most of the relevant points and the causal connectors between them, also showed sensitivity to relevance and more coherence-building strategies during the course of reading.

Finally, the present study demonstrates the utility of combining online measures of processing, such as eye tracking, with offline measures of comprehension. This kind of methodological triangulation is crucial to understand how the reading processes reflected in eye movements are linked to the memory representation constructed of the text. The present results show that good comprehension of text, as measured by the quality of the oral summary produced after reading, is related to a selective processing strategy during reading. Thus, the present study adds to the current literature important knowledge about what kind of processing strategies are successful in that they result in good text comprehension.

### Critical evaluation of the present study

The present results are in line with previous studies showing that task-relevance increases processing effort and subsequent recall of text information [ 21, 25, 26, 8, 9, 16 ], and expand these earlier findings by showing that there are individual differences in how readers make use of this selective attention strategy. Unfortunately, on the basis of the current data, it is impossible to say what the underlying factor for these differences is. In some previous studies readers with high working memory capacity showed greater relevance effects on text recall [ 23 ] and in processing [ 21 ], suggesting that working memory capacity might be a crucial factor in how efficiently readers can make use of the selective attention strategy. However, these previous studies did not directly examine how the quality of the recall was related to processing, as was done in the present study. Future studies should examine in more detail the factors that influence a reader’s ability to selectively attend to and to build a coherent memory representation of relevant text information.

The eye tracking methodology used in the present study did not allow us to examine processing on a word or sentence level – only paragraph-level measures and transitions between paragraphs could be analyzed. While paragraph level analysis proved to be good enough for understanding what kind of strategies different readers used during the course of reading, more accurate measurements of eye movements would provide valuable information on whether the individual differences emerge already at the levels of word processing or sentence reading. These types of analyses might be helpful in understanding the underlying factors of the individual differences observed in summarization skills.

### Conclusions

In line with current theories of text comprehension [ 7, 4, 5 ], the present study shows that when readers are given a task, they try to form a coherent memory representation of the text in which task-relevant information is prioritized. This is done by utilizing a selective attention strategy: extra processing effort is directed to task-relevant text information. Readers who demonstrate good summarization skills show increased sensitivity to task-relevance by spending extra time on relevant text information already during first-pass reading, and they also tend to look back from the question-relevant paragraph to the paragraph containing the question, implying that they try to build links between the question and question-relevant text information. We suggest that this coherence-building activity is reflected in the high-quality summaries of these readers.

The present results demonstrate that presenting a question in the beginning of a text helps some readers to build a coherent summary of the text information. However, not all readers are capable of utilizing a selective attention strategy efficiently. These individual differences may be related to general cognitive constraints, such as working memory capacity [ 23, 21 ], or knowledge about efficient processing strategies [ 52 ] (Hyönä & Nurminen, 2003). Future studies should examine in more detail the conditions in which presenting questions in the beginning of text support comprehension, as this would be valuable information for developing efficient educational practices.

Finally, the present results suggest that a summarization task is a useful pedagogical tool, as it is likely to increase elaborative processing and coherence-building activities during the course of reading (43). Moreover, the results show that the quality of the summary reflects the degree to which the reader was engaged with these types of activities. Thus, the summarization task serves as a comprehension-enhancing intervention and the summary itself provides some information about the processes that occurred during the course of reading. In the context of learning, this information could be used to give feedback to the learners about the efficiency of their reading strategies.

## Ethics and Conflict of Interest

The author(s) declare(s) that the contents of the article are in agreement with the ethics described in http://biblio.unibe.ch/portale/elibrary/BOP/jemr/ethics.html and that there is no conflict of interest regarding the publication of this paper.

## Acknowledgements

We are thankful to all the Turku EyeLabs people for their support, help, and advice during the accomplishment of this study, and specifically to professor Jukka Hyönä for his insightful comments and support.

## Funding

The work reported in this manuscript was supported by Grant PSI2013-47219-P from the Ministry of Economic and Competitiveness (MINECO) of Spain.

## Appendix

### Appendix A

One of the experimental texts, the coding scheme for this text and some summary examples:

The Thames

For centuries, London has been exposing the Thames to high levels of contamination. In 1849 it was found that salmon, like the rest of the flora and fauna, had disappeared from the river. The water, though, was still used for human consumption, a fact which led to over 35,000 deaths from diphtheria epidemics between 1831 and 1866. But how did the river become so contaminated?

Because London was a large, heavily populated and industrialized city, the pollution dumped into the river was of a mixed nature. First, the Thames received huge amounts of untreated organic waste from the sewers of London. Second, industries produced chemical waste (such as hydrocarbons, synthetic detergents, phenols, cyanide) that changed the pH of the water. Both types of pollution completely extinguished any form of life in the river.

The contamination led Londoners to avoid the Thames in summer. Every viscous drop of water that passed carried the smell of two centuries of urban pollution. And beneath the surface, the river was dead. In more than 70 kilometers, the water contained almost no oxygen, and fish and other living creatures that inhabited the river had been eliminated long ago. Until the 80´s, the Thames was one of the most polluted rivers in the world.

Coding scheme for “The Thames” text:

Two relevant factors: organic waste from houses and chemical waste from industries.

0 points. No mention is made about the factors or their causal connections.

1 point. One of the factors without causal connection: not all the factors (chemical or organic residues) are mentioned or it is mentioned that there are two factors but without specifying which ones. In addition, it is not specified that the factors mentioned are the cause of river pollution.

2 points. One of the factors with causal connection: not all the factors (chemical or organic residues) are mentioned. However, it is specified that the factor mentioned is the cause of river pollution.

3 points. The two factors without causal connection: both factors (chemical and organic residues) are explicitly mentioned, but it is not explicit that they are the cause of the contamination of the river.

4 points. The two factors with causal connection: both factors (chemical and organic residues) are explicitly mentioned, and it is also made explicit that they are the cause of the contamination of the river.

HQS Summaries:

“Their contamination was mixed, on the one hand due to the organic residues that were poured by the Londoners' drains and on the other hand due to the chemical residues that changed pH of the water”.

“The River Thames became contaminated because London was a heavily industrialized city, and it was contaminated in two ways: firstly, by organic waste, London's sewer areas were not properly purged and waste was thrown directly into the River Thames, spoiling the water. On the other hand, all the factories poured contaminating products in the River Thames like hydrocarbons, cyanides and others. And that changed the pH of the water and caused the river to become polluted”.

MQS Summaries:

“The river was contaminated by chemical waste, especially chemicals and also they used the river as the place where the drainage and other residues ended, which in the end made disappear the fauna and flora of the river”.

“The river was polluted by organic sewage from all over London and by chemical waste from factories”.

LQS Summaries:

“It is more polluted because there is not enough oxygen in the deepest, and this is because they have made lot of toxic waste and due to remove oxygen”.

“Due to industrialization and that there was no efficient depuration”.

### Appendix B

Instructions for participants:

Participants were told: “You will read a set of short expository texts. We want you to read the text carefully, focus on the question that appears at the end of the first paragraph, and try to understand as much of the text as possible to answer the question. Later, after reading, you will be asked to give an oral summary about the main ideas of the text including information related to the question to see how well you understood what you have read”.

### Appendix C

**Table 1:** Model for the total fixation time

Random effects			
Group	Variance	SD	
Participant	13.09	3.62	
Text	.68	.82	
			
**Fixed effects**			
	b	95% CI	*t*
Intercept	5.75	[3.39 - 8.11]	4.78
Relevance	3.00	[1.41 - 4.58]	3.70
MQS Group	-.80	[-4.00 - 2.41]	-.49
HQS Group	1.42	[-1.72 - 4.56]	.89
Relevance*MQS	1.03	[-1.21 - 3.28]	.90
Relevance*HQS	6.98	[4.78 - 9.18]	6.21

**Table 2:** Model for the first-pass reading time

Random effects			
Group	Variance	SD	
Participant	5276566	2297.1	
Text	430886	656.4	
			
**Fixed effects**			
	b	95% CI	*t*
Intercept	5374.6	[3769.79 – 6979.31]	6.56
Relevance	871.5	[-349.42 – 2092.45]	1.40
MQS Group	-840.9	[-2987.79 – 1305.98]	-.77
HQS Group	381.6	[-1724.20 – 2487.46]	.36
Relevance*MQS	1432.2	[-294.44 – 3158.87]	1.63
Relevance*HQS	2306.8	[611.49 – 4002.20]	2.67

**Table 3:** Model for the look-back duration

Random effects			
Group	Variance	SD	
Participant	4820989	2196	
Text	0	0	
			
**Fixed effects**			
	b	95% CI	*t*
Intercept	375.55	[-1142.41 – 1893.51]	0.49
Relevance	2133.68	[802.75 – 3464.60]	3.14
MQS Group	48.29	[-2101.26 – 2197.83]	.04
HQS Group	1040.69	[-1067.35 – 3148.72]	.97
Relevance*MQS	-409.82	[-2292.04- 1472.39]	-.43
Relevance*HQS	4658.45	[2810.38 – 6506.53]	4.94

**Table 4:** Model for the number of returns to the introductory paragraph

Random effects			
Group	Variance	SD	
Participant	.15	.38	
Text	.002	.05	
			
**Fixed effects**			
	b	95% CI	*t*
Intercept	.35	[.06 - .63]	2.39
Relevance	.26	[-.008 - .53]	1.90
MQS Group	.003	[-.40 - .40]	.01
HQS Group	.13	[-.25 - .52]	.66
Relevance*MQS	.08	[-.30 - .46]	.42
Relevance*HQS	.72	[.34 - 1.10]	3.76

**Table 5:** Model for the duration of look-backs to the introductory paragraph

Random effects			
Group	Variance	SD	
Participant	482721	694.8	
Text	207730	455.8	
			
**Fixed effects**			
	b	95% CI	*t*
Intercept	892.21	[158.26 – 1626.15]	2.38
Relevance	-146.87	[-874.65 – 580.91]	-.40
MQS Group	-31.50	[-934.25 - 871.24]	-.07
HQS Group	-59.71	[-944.23 – 824.82]	-.13
Relevance*MQS	-136.80	[-1166.04 – 892.43]	-.26
Relevance*HQS	1062.67	[52.09 – 2073.24]	2.06

**Table 6:** Model for the summary task performance (word count)

Random effects			
Group	Variance	SD	
Participant	19.18	4.38	
Text	4.60	2.15	
			
**Fixed effects**			
	b	95% CI	*t*
Intercept	.39	[-3.45 – 4.22]	.20
Irrelevant	1.09	[-2.39 – 4.58]	.61
Relevant	19.51	[16.01 – 23.01]	10.93
MQS Group	2.50	[-2.33 – 7.33]	1.02
HQS Group	2.83	[-1.91 – 7.57]	1.17
Irrelevant*MQS	-2.08	[-6.98 – 2.81]	-.83
Irrelevant*HQS	-.93	[-5.73 – 3.87]	-.38
Relevant*MQS	12.78	[7.89 – 17.68]	5.12
Relevant*HQS	31.55	[26.74 – 36.37]	12.84
